# Severity and Longitudinal Course of Depression, Anxiety and Post-Traumatic Stress in Paediatric and Young Adult Cancer Patients: A Systematic Review and Meta-Analysis

**DOI:** 10.3390/jcm12051784

**Published:** 2023-02-23

**Authors:** Ainsley Ryan Yan Bin Lee, Chun En Yau, Chen Ee Low, Jiaqi Li, Roger C. M. Ho, Cyrus Su Hui Ho

**Affiliations:** 1 Yong Loo Lin School of Medicine, National University of Singapore, Singapore 119228, Singapore; 2 School of Clinical Medicine, University of Cambridge, Cambridge CB2 1TN, UK; 3 Department of Psychological Medicine, Yong Loo Lin School of Medicine, National University of Singapore, Singapore 119228, Singapore; 4 Department of Psychological Medicine, National University Hospital, Singapore 119228, Singapore

**Keywords:** depression, anxiety, post-traumatic stress, childhood cancer, paediatric cancer, psychosocial oncology, supportive care in cancer, meta-analysis

## Abstract

Background: A diagnosis of cancer and treatment may constitute a highly traumatic period for paediatric cancer patients (PYACPs). However, no review has comprehensively analysed how the mental health of PYACPs is acutely affected and the longitudinal course. Methods: This systematic review followed PRISMA guidelines. Comprehensive searches of databases were conducted to identify studies of depression, anxiety and post-traumatic stress symptoms in PYACPs. Random effects meta-analyses were used for the primary analysis. Results: From 4898 records, 13 studies were included. Acutely after diagnosis, depressive and anxiety symptoms were significantly elevated in PYACPs. Depressive symptoms only significantly decreased after 12 months (standardised mean difference, SMD = −0.88; 95% CI: −0.92, −0.84). This downward trajectory persisted to 18 months (SMD = −1.862; 95% CI: −1.29, −1.09). Anxiety symptoms similarly only decreased after 12 (SMD = −0.34; 95% CI: −0.42, −0.27) up to 18 months (SMD = −0.49; 95% CI: −0.60, −0.39) after the cancer diagnosis. Post-traumatic stress symptoms showed protracted elevations throughout follow-up. Overall, significant predictors of poorer psychological outcomes included unhealthy family functioning, concomitant depression or anxiety, poor cancer prognosis or experiencing cancer and treatment-related side effects. Conclusions: While depression and anxiety may improve over time with a favourable environment, post-traumatic stress may have a protracted course. Timely identification and psycho-oncological intervention are critical.

## 1. Introduction

The incidence and global burden of cancer have steadily increased in the twenty-first century and remain one of the top causes of mortality. Amongst these, there are estimated to be over 300,000 new diagnoses a year in paediatric and young adult cancer patients (PYACPs), with the most common cancers being leukaemias, brain cancers and lymphomas [1,2]. With the development and progression of antineoplastic therapy, survival in patients with paediatric cancers has risen over the years [3,4]. However, psychological stress and trauma remain significant sources of morbidity in PYACPs and their family unit [5,6,7].

This psychological burden may stem from various dimensions, including the fear of death, pain from disease and treatment and the stress it places on those around them. In particular, cancer and its associated treatment may result in persistent symptoms such as pain and fatigue and medical comorbidities, resulting in poorer quality of life [7,8]. Following diagnosis, immense disruption to the life of patients may also occur, with interruption to schooling, loss of employment and reduced social interactions [6,9,10,11]. PYACPs, thus, suffer disproportionately not only from physical complications related to cancer and its treatment [12], but from psychological symptoms and disorders as a result [13]. As PYACPs are in their formative years [5], the psychological response may involve negative feelings of depression, anxiety and post-traumatic stress symptoms (PTSS). If unaddressed, this high level of PTSS may translate to a greater risk of psychiatric sequelae due to their vulnerability to traumatic stress during stages of adolescence and young adulthood [14,15,16]. Overall, survey-based studies found that one in ten paediatric cancer patients displayed severe distress suggestive of trauma five to six weeks after diagnosis [17]. This highlights a period of particular vulnerability at which intervention would be opportune. As such, examining the trajectory of psychological symptoms following a cancer diagnosis is critical.

Few distinct cohort studies over the years have highlighted varying trajectories of the course of psychological disorders and symptoms in childhood, adolescents and young adult cancer patients and survivors. Overall, detailed data on the course of these symptoms are limited. Furthermore, no systematic review has sought to analyse the trajectory of PYACPs as a whole. Elucidating the source of depression, anxiety and PTSS would be valuable in identifying periods of vulnerability during which psychological support and intervention would be most opportune.

## 2. Materials and Methods

### 2.1. Protocol and Guidance

The systematic review is reported according to the Preferred Reporting Items for Systematic Reviews and Meta-Analyses (PRISMA) guidelines [18] without prospective registration.

### 2.2. Data Sources and Search Strategy

A literature search was performed in PubMed, MedLine, Embase and PsycINFO from inception to December 2022. The search strategy combined search terms for paediatrics, children, adolescents, cancer, depression, anxiety and post-traumatic stress symptoms. The database-controlled vocabulary was used for searching subject headings, and a large spectrum of synonyms with appropriate truncations was used for searching title, abstract and author keywords. Searches of the reference lists of all relevant articles was also performed to identify any additional articles. The search strategy was translated between each database. Examples of the full strategies for PubMed and EMBASE are available in Table S1.

### 2.3. Study Selection: Inclusion and Exclusion Criteria

Two reviewers (A.R.Y.B.L. and C.E.Y.) independently screened titles and abstracts of all studies for eligibility according to the inclusion and exclusion criteria. The full text of studies assessed as ‘relevant’ or ‘unclear’ was then independently evaluated by two reviewers (A.R.Y.B.L. and C.E.Y.). The inter-rater agreement was computed, and discrepancies were resolved by adjudication by a third independent reviewer (C.E.L.).

In our review, paediatric and young adult cancer patients (PYACPs) are defined as those no older than 25 years old at the point of cancer diagnosis, following the definition of the World Health Organisation. Their family unit includes their siblings, parents, caregivers or other individuals living in the same household as the PYACP. We included studies that recruited and reported psychological outcomes in PYACPs. If studies included patients with cancer older than 25 years old at the time of diagnosis, the study would be excluded if they did not report outcomes of PYACPs separately and no response was received from the corresponding authors to provide these data by the time of analysis in January 2023.

The primary outcomes were the change in severity of depressive, anxiety and post-traumatic stress symptoms in PYACPs over time after the diagnosis of cancer. The secondary outcomes were the severity of depressive, anxiety and post-traumatic stress symptoms in PYACPs in comparison to a non-cancer control group, and any risk factors. We included studies that assessed and reported symptom scores of depression, anxiety and PTSS over at least two timepoints after the diagnosis of cancer, thus allowing the longitudinal change to be assessed. We only included prospective follow-up studies published in peer-reviewed journals as full-text articles.

### 2.4. Data Extraction

Two reviewers (A.R.Y.B.L. and C.E.Y.) independently extracted data from each included article according to a predefined structured proforma in Microsoft Excel Version 16.64. Additional quality checking of data was performed at the end of the extraction stage prior to data analysis. Study data included the type of study, year of publication, location of study, recruitment methodology, follow-up duration and data related to participant loss to follow-up or drop out. Participant data included the age, sex, type of cancer, treatment received and comorbidities. Outcome-related data included the baseline and outcome scores at each timepoint when outcomes were assessed, instrument and method used to assess outcomes and the effect of mediating factors related to cancer and treatment-related factors, family and environmental factors, patient factors and social and economic factors. The effect sizes, *p* values and confidence intervals of analysis, such as univariate or multivariate regression, to determine the mediating effect of factors reported in each study, were extracted.

### 2.5. Risk of Bias Assessment

To assess methodological quality and risk of bias of studies, the Joanna Briggs Institute (JBI) Critical Appraisal checklist [19], which includes appraisal of the criteria for inclusion, measurement of condition, reporting of baseline characteristics, reporting of outcomes and appropriateness of the statistical analysis, if any was used [20]. This appraisal was performed by two reviewers (A.R.Y.B.L. and C.E.Y.) independently, with discrepancies resolved by an independent verdict of a third reviewer (C.E.L.). The maximum score attainable, signifying the highest quality, was 11 points for cohort studies.

### 2.6. Data Analysis

We conducted all analyses on R (version 4.1.0) using the *meta* and *metafor* packages. Unless otherwise specified, we considered a two-sided *p* value of <0.05 as statistically significant. For continuous outcomes, in studies without standard deviations (SDs), confidence intervals (CIs) were converted to SDs. Studies were pooled for meta-analysis using standardised mean differences (SMD). Sensitivity analysis was conducted using random-effects, common-effects and leave-one-out analyses, and the identification and exclusion of potential outliers. Between-study heterogeneity was represented by I^2^ and τ^2^ statistics. An I^2^ of <30% indicated low heterogeneity between studies, 30% to 60% showed moderate heterogeneity, and >60% indicated substantial heterogeneity [21]. Studies were pooled according to the amount of time elapsed since the diagnosis of cancer in PYACPs.

To study the acute psychological reaction in PYACPs, symptom scores were pooled in cohorts of PYACPs within a month of diagnosis of cancer, and the level of psychological symptoms compared to non-cancer controls. A positive SMD signified a greater severity of psychological symptoms compared to non-cancer controls, and a negative SMD signified a lesser severity of psychological symptoms compared to non-cancer controls. To evaluate the longitudinal trend of psychological symptoms after diagnosis, studies which assessed the severity of psychological symptoms in the same cohort of PYACPs at multiple time points after the diagnosis of cancer were included. A positive SMD signified a greater severity of psychological symptoms from baseline, and a negative SMD signified a lesser severity of psychological symptoms from baseline. To calculate the standardised means, we used the *escalc* function in the *metafor* package. In this package, the positive bias in the standardised mean difference (i.e., in a Cohen’s d value) is automatically corrected for within the function, yielding Hedges’ g.

As we expected significant heterogeneity in the measurement, reporting and definitions of risk, protective and exacerbating factors such as degree of family functioning and socioeconomic status, we planned to use the synthesis without a meta-analysis approach [22]. These factors were categorised as exacerbating factors if they significantly increased the risk or severity of psychological symptoms, or protective factors if they significantly decreased the risk or severity of psychological symptoms. If the association did not reach statistical significance, this was extracted and reported.

We assessed for publication bias both qualitatively, via visual inspection for funnel plot asymmetry, and quantitatively, using Egger’s test. Where publication bias was suspected based on either Egger’s regression intercept test of bias or visual inspection of funnel plot asymmetry, we conducted a sensitivity analysis using the trim-and-fill method (R0 estimator, fixed-random-effects models) to re-estimate the pooled effect size after imputing potentially missing studies [23,24]. This assumes a normal distribution of effect sizes around the centre of the funnel plot if publication bias is absent [25].

## 3. Results

**Figure 1:**
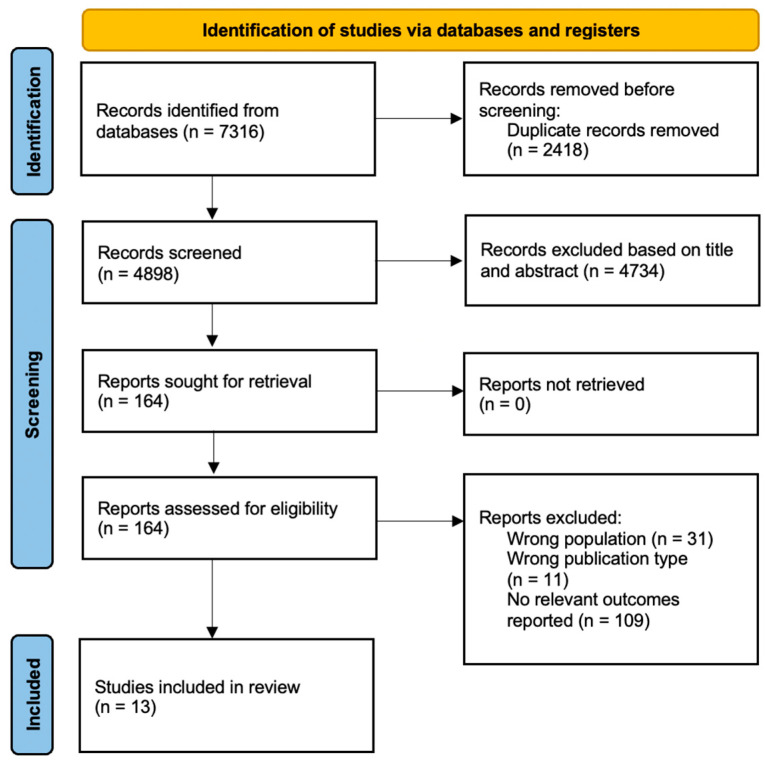
PRISMA flowchart.

From 4898 records, we included a total of 13 studies [26,27,28,29,30,31,32,33,34,35,36,37,38] (Figure 1) with the key characteristics reported in Table 1. Inter-rater agreeability as measured by the Kappa statistic was high, with 0.87 for initial screening based on title and abstracts and 0.94 for review of full texts.

### 3.1. Longitudinal Course of Depressive Symptoms after Diagnosis of Cancer

**Figure 2:**
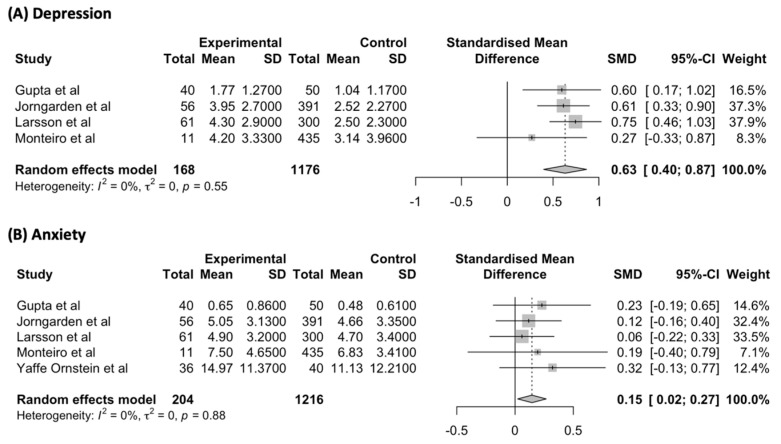
(**A**) Depressive symptoms in PYACPs acutely within a month of cancer diagnosis compared to non-cancer comparators; (**B**) Depressive symptoms in PYACPs acutely within a month of cancer diagnosis compared to non-cancer comparators. Standardised mean difference, SMD; standard deviation, SD; 95% confidence interval, 95% CI [29,30,37,38,39].

A meta-analysis of studies assessing the severity of depressive symptoms acutely, within a month of the diagnosis of cancer, compared to matched non-cancer comparators, was performed [29,30,37,38] (Figure 2A). Depressive symptoms were found to be significantly more severe in PYACPs compared to non-cancer comparators, with minimal heterogeneity between studies (SMD = 0.63; 95% CI: 0.40, 0.87; I^2^ = 0%).

**Figure 3:**
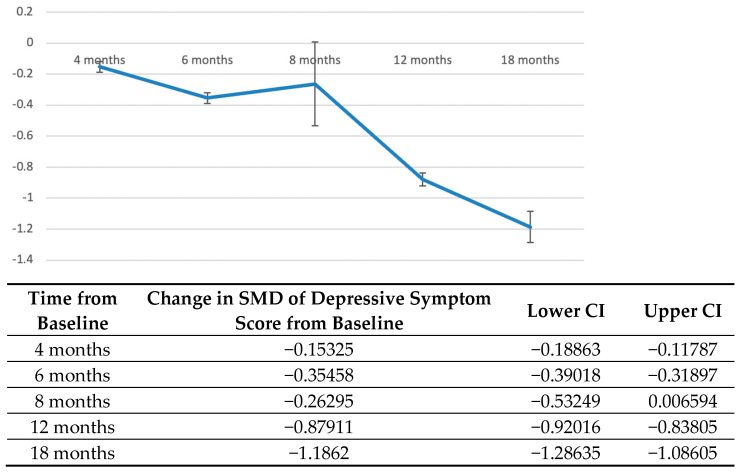
Longitudinal course of depressive symptoms in PYACPs after diagnosis. Vertical axis: Standardised mean difference in depressive symptom scores from baseline, with a negative value representing a decrease. Horizontal axis: Time elapsed from diagnosis in months. Standardised mean difference, SMD; confidence interval, CI.

A further analysis of studies assessing the course of depressive symptoms in PYACPs was performed [26,27,28,29,30,31,34] (Figure 3). As different scales were used across studies (Table S2), meta-analysis of standardised means was performed in comparison to symptoms measured after the diagnosis of cancer.

Overall, a decreasing trend was identified over time. Depressive symptoms remained elevated in studies up to eight months after the diagnosis of cancer, before a sharp and significant decrease in SMD of depressive symptom score by 12 months (SMD = −0.88; 95% CI: −0.92, −0.84). This decrease persisted until 18 months (SMD = −1.862; 95% CI: −1.29, −1.09).

Of the included studies, three compared the longitudinal course of depression in paediatric cancer patients with their parents [28,31,34]. Sargin Yildirim et al. [28] found mean anxiety and depression subscale scores statistically significantly higher during treatment relative to those estimated before and at the end of treatment (all *p* < 0.01). Based on depression subscale scores, a significantly higher number of patients with depressive symptoms was detected during treatment (36%) when compared with before (18%) and at the end of treatment (14%). Yardeni et al. [31] compared the severity of depressive symptoms and prevalence of depressive disorder in children with cancer and their parents from one to twelve months following the diagnosis. There was a significant decrease in both the mean depression symptom score and prevalence of depressive disorders in both paediatric patients and their parents over time. Prikken et al. [34] found a largely similar trend.

Four studies following up a cohort of PYACPs over the period of treatment compared depressive symptoms to a reference cohort with no personal relation to the PYACPs being treated [26,27,29,30]. Jorngarden et al. [29] and Larsson et al. [30] both found that depressive symptoms worsened after the time of diagnosis, being significantly more severe than the reference cohort. Both studies found that depressive symptoms were similar to the reference cohort 18 months after diagnosis. However, Kunin-Batson et al. [26] found that the proportion reporting clinically significant depressive symptoms remained fairly consistent over the course of cancer treatment and after completing treatment. Myers et al. [27] found the frequency of depression scores in the clinically significant range was not significantly different from expected levels at any timepoint.

### 3.2. Risk, Protective and Exacerbating of Depressive Symptoms

The risk, protective and exacerbating of depressive symptoms were synthesised and reported in Table S2. Amongst studies which compared the effect of having a central nervous system cancer against other cancers, a significant association was found with higher depressive symptoms [28,33]. In terms of the patient’s family unit and environment, having healthy family functioning was consistently found to be a significant predictor of an improved course of depressive symptoms over time [26,27]. If the parents of the patient were not married over the course of cancer, Myers et al. [27] reported there to be significantly higher depressive symptoms, while Kunin-Batson et al. found no significant association [26]. Patients who experienced concomitant anxiety [31] or had poorer physical function were significantly more likely to have higher depressive symptoms.

### 3.3. Longitudinal Course of Anxiety Symptoms after Diagnosis of Cancer

Meta-analysis of studies assessing the severity of anxiety symptoms acutely, within a month of the diagnosis of cancer, compared to matched non-cancer comparators, was performed [29,30,37,38,39] (Figure 2B). As different scales were used across studies (Table S2), a meta-analysis of standardised means was performed in comparison to symptoms measured after the diagnosis of cancer. Anxiety symptoms were found to be significantly more severe in PYACPs compared to non-cancer comparators, with minimal heterogeneity between studies (SMD = 0.15; 95% CI: 0.02, 0.27; I^2^ = 0%).

**Figure 4:**
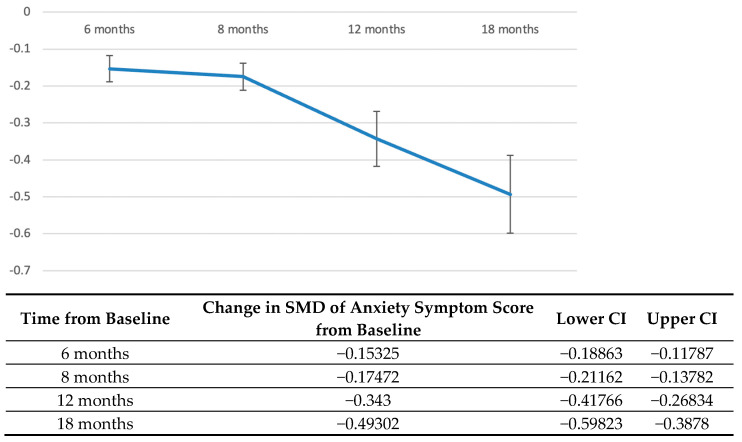
Longitudinal course of anxiety symptoms in PYACPs after diagnosis. Vertical axis: Standardised mean difference in anxiety symptom scores from baseline, with a negative value representing a decrease. Horizontal axis: Time elapsed from diagnosis in months. Standardised mean difference, SMD; confidence interval, CI.

A further analysis of studies which longitudinally assessed the course of anxiety symptoms was performed [26,27,28,29,30,31] (Figure 4). Just like with depressive symptoms, anxiety symptoms also demonstrated a decreasing trend over time. Anxiety symptoms again only demonstrated a decrease in SMD by 12 months (SMD = −0.34; 95% CI: −0.42, −0.27), continuing to decrease until 18 months (SMD = −0.49; 95% CI: −0.60, −0.39).

Two studies compared anxiety in PYACPs and their parents. Sargin Yildirim et al. [28], similar to depressive symptoms, found statistically significantly higher scores during treatment relative to those estimated before and at the end of treatment. In contrast, Yardeni et al. [31] found that anxiety symptom scores and prevalence of anxiety disorders in both paediatric patients and their parents over time remained relatively constant.

Four studies following up a cohort of PYACPs over the period of treatment compared anxiety symptoms to a reference cohort with no personal relation to the PYACPs being treated [26,27,29,30]. All four studies had similar findings, with the prevalence of severe anxiety and mean anxiety scores rising following treatment before decreasing to a level insignificantly different from the comparator cohort.

### 3.4. Risk, Protective and Exacerbating of Anxiety Symptoms

The risk, protective and exacerbating of anxiety symptoms are reported in Table S2. Amongst studies which compared the effect of having a central nervous system cancer against other cancers, a significant association was again found with higher anxiety symptoms [28,33]. Experiencing more cancer-related pain or being in the acute phase of treatment was associated with higher anxiety [31], but the type of treatment received was not [33]. Unlike depressive symptoms, the health of family functioning and marriage status of parents had varying significance across studies. Patients who experienced concomitant depression [31] or had poorer physical function were significantly more likely to have higher anxiety symptoms.

### 3.5. PTSS after Diagnosis of Cancer

Two studies evaluating post-traumatic stress were identified [32,35]. Due to limited longitudinal data, meta-analysis was not performed. Werk et al. and Kwak et al. studied PTSS up to approximately 12 months after the diagnosis of cancer. Werk et al. recruited a large cohort of 1721 PYACPs with Hodgkin’s lymphoma, while Kwak et al. recruited a smaller cohort of 87 PYACPs with various cancers. Both studies found that levels of PTSS remained relatively consistent up to 12 months after diagnosis.

Kwak et al. found those with a poorer cancer prognosis had significantly higher PTSS at all time points; however, Werk et al. found no association of stage of disease with course of PTSS. The findings of Werk et al. may be explained by the entire cohort of PYACPs having Hodgkin’s lymphoma, a high proportion of over 80% of whom were rapid responders to treatment. The subgroup of PYACPs who experienced a relapse of the disease did indeed have significantly higher odds of elevated PTSS. Experiencing greater symptomatology, activity limitations or a disruption in employment or schooling also predicted higher PTSS, but no association was found with sex or age at diagnosis.

### 3.6. Risk of Bias

The risk of bias assessment performed using the Joanna Briggs Institute Critical Appraisal checklist is reported in Table S3. As psychological symptoms could not be determined before the diagnosis of cancer in all studies, PYACPs could not feasibly be classified as being free of the outcome at inclusion in the study, thus this domain was scored as ‘not applicable’. However, all studies assessed psychological symptoms at a baseline time-point, allowing the trajectory of symptoms to be evaluated, and are thus not at risk of bias. Only Werk et al. [32] and Kwak et al. [35] did not include a control group. Overall, no studies presented a significant risk of bias. The assessment of publication bias was performed with funnel plots and trim-and-fill plots presented in Figures S1–S4, suggesting some publication bias may be present.

## 4. Discussion

To the best of our knowledge, this systematic review and meta-analysis is the first to comprehensively analyse the longitudinal course of depression, anxiety and post-traumatic stress amongst paediatric and young adult patients with cancer. In children and adolescents currently undergoing antineoplastic treatment, both depression and anxiety demonstrated similar trends, with a significant exacerbation in symptoms within the first month to a year from diagnosis, before having a downward trajectory. However, the burden of depressive symptoms may remain elevated without decreasing to pre-diagnosis levels.

The phenomenon of amelioration in depressive and anxiety symptoms by 12 months may be explained by post-traumatic growth after the acute period of trauma [40]. Post-traumatic growth, considered a positive change in the psyche following struggling with highly stressful life circumstances, is a phenomenon particularly described following a cancer diagnosis [41,42]. It has also been demonstrated in other forms of trauma, including traffic accidents or losing loved ones [43,44]. In stark contrast, those who experienced significant post-traumatic growth, in a number of studies, reported lower levels of depression and anxiety than the general population [45,46,47]. As such, post-traumatic growth and maturity may contribute to a conversely reduced psychological burden over time [48].

This review also identified critical mediating factors that may result in higher psychological symptoms and a poorer trajectory over time. A healthy family environment was consistently found to be a strong and significant protective factor against depressive and anxiety symptoms. The importance of a positive environment and interactions following the period of trauma were similarly highlighted in previous studies to be significant contributors to post-traumatic growth and positive psychological prognosis [44,49,50,51]. Psycho-oncological interventions may also seek to capitalise on modifiable exacerbating and protective factors, as highlighted in this review. With the significant influence family functioning has on the trend of depressive symptoms, interventions may seek to improve cohesiveness and support for PYACPs involving the family unit. This may also be complemented by appropriate pharmacological and adjunctive treatments for psychological comorbidities [52,53,54].

The importance of supportive care to alleviate the burden of cancer is also critical. Experiencing more severe cancer and treatment-related symptomatology such as pain and fatigue was strongly associated with poorer psychological outcomes. Those who experienced adverse life events, such as disruptions in employment or schooling, were also more likely to have persistent depression and anxiety. Thus, it is crucial to recognise the burden cancer and its associated treatment may have, and provide due support to those who suffer from it in order to reduce the lasting psychological impact.

The findings in this review revealed that PTSS manifested as early as six months post-diagnosis and was relatively consistent up to 12 months after. This finding is consistent with another study by Phipps et al. [55], in which similar levels of PTSS were observed in childhood cancer survivors up to 18 months after diagnosis. However, the study by Phipps et al. did not follow up the same cohort of childhood cancer survivors, instead following up four separate cohorts interviewed at different points in time. Nonetheless, prior studies of long-term survivors of childhood cancer that used various methods to determine post-traumatic stress disorder have reported rates ranging from under less than one in twenty to more than one in five [56,57]. These findings highlight the potential value of opportunistic screening and early intervention for PTSS among PYACPs during the first year following diagnosis. Such interventions may involve tailored psychotherapeutic and behavioural elements to capitalise on protective factors, such as the family unit [58,59]. By addressing and alleviating acute trauma, this may reduce the risk of long-term post-traumatic stress disorder and its associated morbidity in PYACPs.

In the studies included in this review, studies that recruited siblings or parents of the PYACPs allowed valuable insights into the trajectory of psychological symptoms within the family unit. Just like the PYACPs, siblings or parents of patients with childhood cancer can experience psychological stress after an event. This burden may stem from worry for the patient with cancer, alterations in family dynamics or socioeconomic consequences of the diagnosis and treatment [60]. Another large cohort study of 2645 PYACPs and their siblings found that the prevalence of depression was almost doubled, at 15% of siblings reporting depression, exceeding the 8% prevalence in survivors [61]. Similarly, studies included in our review found that the level of psychological symptoms in PYACPs, their siblings and their parents were often insignificantly different from each other, but higher than normative population values.

The findings of our review highlight several pertinent research gaps future studies may seek to address. No studies were identified which involved the follow-up of psychological symptoms in PYACPs more than two years from the point of diagnosis. There is, thus, a lack of information about the longer-term trajectory. Knowledge of protective and exacerbating factors of post-traumatic stress symptoms is also lacking, with only two studies reporting it [32,35]. As a whole, the included studies did not have sufficient sample sizes for the mediating effect of cancer treatment-related factors to be elucidated. For example, the short- and long-term adverse events of systemic chemotherapy vary greatly between drugs used.

Our review faced several limitations. There is a distinct paucity of studies that evaluated the longitudinal course of depression, anxiety and post-traumatic stress following the diagnosis of cancer. Secondly, there was a degree of heterogeneity in the instruments and questionnaires for quantifying the symptom burden of psychiatric disorders. While all instruments used are widely validated and evaluate similar domains, there remains heterogeneity in the assessment that may not be accounted for. Thirdly, studies spanned a range of countries, and sociocultural and economic backgrounds. This may further vary across countries and populations studied, which we were unable to plan statistical pooling for. Instead, we adopted the synthesis without a meta-analysis approach. Fourthly, we did not obtain individual patient data for our meta-analysis, which may result in the assessment of risk factors being less granular. We overcame this by systematically synthesising the individual analyses performed by each study to identify vulnerability factors.

## 5. Conclusions

A diagnosis of cancer and its treatment may result in significant psychological trauma and disruption to the life of patients and their family unit. Symptoms of depression and anxiety were significantly elevated following the diagnosis, but exhibited a downtrending course from 12 months. However, post-traumatic stress symptoms may remain elevated, representing a pertinent psychological comorbidity to be cognisant of. Dedicated assessment for post-traumatic stress in PYACPs is warranted. Risk factors predicting a poorer psychological prognosis such as health of family functioning, social environment and cancer and cancer treatment-related symptomatology were identified. Overall, elucidating the trajectory of symptoms allows us to identify populations at exceptional vulnerability that would benefit from timely identification and psycho-oncological intervention.

## Figures and Tables

**Table 1:** Characteristics of included studies.

Author	Publication Year	Region of Study	N, Cancer Type	Control Characteristics	Age at Cancer Diagnosis *	Age at the Time Data Were Collected *	Depression Scale	Anxiety Scale	Post-Traumatic Stress Scales
Kunin-Batson et al. [26]	2016	USA	160, Acute lymphocytic leukaemia	Normative population data	Range: 1–9.9	Range: 2–10.9	BASC-2	BASC-2	-
Myers et al. [27]	2014	USA	159, Acute lymphocytic leukaemia	Normative population data	4.9 (2.2)	5.9 (2.2)	BASC-2	BASC-2	-
Gupta et al. [38]	2014	India	40, Acute lymphocytic leukaemia	Matched controls	Range: 6 to 14	Range: 6 to 14	CPMS	CPMS	-
Werk et al. [32]	2022	USA	1721, Hodgkin’s lymphoma	No reference group	14.6 (3.3)	14.5 (3.4)	-	-	Novel scale
Kaplan et al. [36]	1987	USA	21, Haematological cancers	Matched controls	9.71 (1.52)	15.4 (1.82)	CDI and BDI	-	-
Desjardins et al. [33]	2019	Canada	91, Central nervous system	Caregivers	NR	11.21 (2.8)	BASC-2	BASC-2	-
Monteiro et al. [37]	2013	Portugal	11, Various	Matched controls	NR	NR	HADS	HADS	-
Prikken et al. [34]	2022	Belgium	125, Various	Parents	14 to 19 years	NR	CES-D	-	-
Kwak et al. [35]	2013	USA	87, Various	No comparison group	22.7	23.2	-	-	PDS, BSI-18 and SF-36
Sargin Yildirim et al. [28]	2017	Turkey	50, Various	Parents	12.14 (2.97)	12.39 (2.97)	CDI	SCARED	-
Jorngarden et al. [29]	2007	Sweden	56, Various	General population	15.7	NR	HADS	HADS	-
Larsson et al. [30]	2010	Sweden	61, Various	Normative population data	Range: 13–19	4 years after diagnosis	HADS	HADS	-
Yardeni et al. [31]	2021	Israel	99, Various	Parents	NR	13.56 (3.63)	PROMIS and K-SADS	PROMIS and K-SADS	-

Abbreviations: Behavior Assessment System for Children, BASC-2; Children’s Depression Inventory, CDI; Screen for Child Anxiety Related Emotional Disorders, SCARED; Hospital Anxiety and Depression Scale, HADS; Childhood Psychopathology Measurement Schedule, CPMS; Patient-Reported Outcomes Measurement Information System, PROMIS; Center for Epidemiologic Studies Depression Scale, CES-D; Depression and Anxiety Module and Kiddie Schedule for Affective Disorders and Schizophrenia for School-Age Children, K-SADS; Posttraumatic Stress Diagnostic Scale, PDS; Brief Symptoms Inventory-18, BSI-18; Medical Outcomes Study 36-Item Short Form Health Survey, SF-36; Beck’s Depression Inventory, BDI; Children’s Depression Inventory, CDI; Not reported in study, NR; Not studied as outcome, -.* Mean (standard deviation, SD) in years reported unless otherwise specified.

## Data Availability

All analysis was developed using published data. All supplementary material related to this submission is available together with this manuscript.

## Supplementary Materials

The following supporting information can be downloaded at: https://www.mdpi.com/article/10.3390/jcm12051784/s1, Table S1: Search strategy; Table S2: Risk, protective and exacerbating factors of psychological symptoms in PYACPs; Table S3: Quality assessment of included cohort studies using the Joanna Brigg’s Institute Critical Appraisal tool; Figure S1: Funnel plot of baseline depressive symptoms; Figure S2: Trim-and-fill plot of baseline depressive symptoms; Figure S3: Funnel plot of baseline anxiety symptoms; Figure S4: Trim-and-fill plot of baseline anxiety symptoms.
