# Supplementary material for: Severity and Longitudinal Course of Depression, Anxiety and Post-Traumatic Stress in Paediatric and Young Adult Cancer Patients: A Systematic Review and Meta-Analysis
Source: J Clin Med. 2023 Feb 23;12(5):1784. doi: 10.3390/jcm12051784 (PMC10003651; doi:10.3390/jcm12051784)
Supplement: Supplementary file 1 [file jcm-12-01784-s001.zip › jcm-2201677-supplementary.pdf]

Journal of
Clinical Medicine

MDPI

Supplementary Materials

Severity and Longitudinal Course of Depression, Anxiety and Post-Traumatic Stress in Paediatric and Young Adult Cancer Patients: A Systematic Review and Meta-Analysis

Ainsley Ryan Yan Bin Lee [1], Chun En Yau [1], Chen Ee Low [1], Jiaqi Li [2], Roger C. M. Ho [3,4] and Cyrus Su Hui Ho [3,4,*]

[1] Yong Loo Lin School of Medicine, National University of Singapore, Singapore 119228, Singapore
[2] School of Clinical Medicine, University of Cambridge, Cambridge CB2 1TN, UK
[3] Department of Psychological Medicine, Yong Loo Lin School of Medicine, National University of Singapore, Singapore 119228, Singapore
[4] Department of Psychological Medicine, National University Hospital, Singapore 119228, Singapore
* Correspondence: pcmhsh@nus.edu.sg

Table S1: Search strategy

EMBASE

#1	(Pediatric* OR paediatric* OR juvenile* OR "young adult*" OR 'childhood'):ti,ab
#2	"neoplasm"/exp or ('neoplasm' OR 'cancer*' OR 'tumo*' OR 'malign*' OR 'leukemia*' OR 'lymphoma*'):ti,ab
#3	(''depression' OR 'depressive' OR 'anxiety' OR 'anxiety disorder' OR 'post-trauma*' OR 'post trauma*' or 'PTSD')/exp

#1 and #2 and #3 and NOT [medline]/lim

PubMed

#1	("Pediatric*"[Title/Abstract] OR "paediatric*"[Title/Abstract] OR "Young Adult"[Title/Abstract] OR "Child*"[Title/Abstract] OR juvenile*[Title/Abstract] OR infant*[Title/Abstract] OR adolesc*[Title/Abstract])
#2	(neoplas*[Title/Abstract] OR cancer*[Title/Abstract] OR tumo*[Title/Abstract] OR malign*[Title/Abstract] OR leukemia*[Title/Abstract] OR lymphoma*[Title/Abstract]))
#3	("Anxiety"[Mesh] OR "Anxiety Disorders"[Mesh] OR "Post-Traumatic*"[Title/Abstract] OR "PTSD"[Title/Abstract] OR "posttraumatic*"[Title/Abstract] OR "Post Traumatic*"[Title/Abstract] OR "Depress*"[Title/Abstract] OR "Depressive*"[Title/Abstract] OR "Anxi*"[Title/Abstract])

#1 and #2 and #3

Table S2: Risk, protective and exacerbating factors of psychological symptoms in PYACPs.

Study	Duration of study	Cancer and treatment-related factors	Family and environmental factors	Patient factors	Social and economic factors

Depression					
Kunin-Batson et al. [26]	Up to 12 months after diagnosis, and 3 months after completion of chemotherapy	*Having a neurological event on therapy had a significantly higher depression than those without (OR=2.99; p=0.042)	*Family function: 'Unhealthy functioning': Significantly higher depression than 'healthy functioning' (OR=2.62; p=0.024)<br><br>Marital status of parents: 'Unmarried' had insignificantly lower anxiety than 'married'	Age at diagnosis: 'School age (5-9 years)' had insignificantly lower depression than 'preschool (2-4 years)'<br><br>Sex: Males had insignificantly lower depression than females	Annual family income: <$50000 had insignificantly lower depression than >$50000
Myers et al. [27]	Up to 12 months after diagnosis	No relevant mediators studied and reported	*Family function: 'Unhealthy functioning': Significantly higher depression than 'healthy functioning' (OR=2.40; p=0.008)<br><br>*Marital status of parents: 'Not married' had significantly higher depression than 'married' (OR=2.36; p=0.017)	Age at diagnosis: 'School age (5-12 years)' had insignificantly lower depression than 'preschool (2-4 years)'<br><br>Sex: Males had insignificantly lower depression than females	No relevant mediators studied and reported
Sargin Yildirim et al. [28]	During treatment and post-treatment	*During treatment: Patients with central nervous system tumours had significantly higher depression than other cancers (p=0.025)<br><br>Post-treatment: Type of cancer had no association with depression	No relevant mediators studied and reported	No relevant mediators studied and reported	No relevant mediators studied and reported
Yardeni et al. [31]	Up to 12 months after diagnosis	Type of cancer: No association with depression<br><br>Currently receiving acute phase treatment: No association with depression	No relevant mediators studied and reported	*Experiencing concomitant anxiety: Significantly higher depression (b=2.04; p=0.014)	No relevant mediators studied and reported
Desjardins et al. [33]	Up to 8 months after diagnosis	*Having low grade gliomas: Significantly higher depression than other cancers<br><br>Receiving cranial radiation therapy: Insignificant association with depression	No relevant mediators studied and reported	*Female sex: Significantly higher depression than male sex<br><br>Age at diagnosis: Insignificant association with depression	No relevant mediators studied and reported
Anxiety					
Kunin-Batson et al. [26]	Up to 12 months after diagnosis, and 3 months after completion of chemotherapy	Having a neurological event on therapy had an insignificantly lower OR than those without	Family function: 'Unhealthy functioning' had insignificantly higher OR than 'healthy functioning'<br><br>Marital status of parents: 'Unmarried' had insignificantly lower anxiety than 'married'	Age at diagnosis: 'School age (5-9 years)' had insignificantly lower OR than 'preschool (2-4 years)'<br><br>Sex: Males had insignificantly higher OR than females	Annual family income: <$50000 had insignificantly lower OR than >$50000

Myers et al. [27]	Up to 12 months after diagnosis	No relevant mediators studied and reported	*Family function: 'Unhealthy functioning': Significantly higher anxiety than 'healthy functioning' (OR=2.24, p=0.033)<br><br>*Marriage status: 'Not married': Significantly higher anxiety than 'married'	Age at diagnosis: 'School age (5-12 years)' had insignificantly lower OR than 'preschool (2-4 years)'<br><br>Sex: Males had insignificantly lower OR than females	No relevant mediators studied and reported
Yardeni et al. [31]	Up to 12 months after diagnosis	*Having a brain tumour: Significantly higher anxiety than other cancers (b=7.35; p=0.027)<br><br>*Currently receiving acute phase treatment: Significantly higher anxiety (b=2.98; 0.014)	No relevant mediators studied and reported	*Experiencing concomitant depression: Significantly higher anxiety (b=2.04; p=0.014)	No relevant mediators studied and reported
Desjardins et al. [33]	Up to 8 months after diagnosis	*Having low grade gliomas: Significantly higher anxiety than other cancers<br><br>Receiving cranial radiation therapy: Insignificant association with anxiety	No relevant mediators studied and reported	*Female sex: Significantly higher anxiety than male sex<br><br>Age at diagnosis: Insignificant association with anxiety	No relevant mediators studied and reported
Post-traumatic stress symptoms					
Werk et al. [32]	6.7 months after diagnosis and 12.4 months after the first assessment	Stage of cancer: Higher stage had insignificantly higher PTSS<br><br>*Experiencing relapse of cancer: Significantly higher PTSS (OR=2.18; p<0.01)<br><br>*Experiencing fatigue symptoms: Significantly higher OR (p<0.01)<br><br>*Experiencing activity limitations: Significantly higher PTSS (OR=1.05; p<0.01)	No relevant mediators studied and reported	*Having concerns about the future: Significantly higher PTSS (OR=1.13; p<0.01)<br><br>Sex: Males had insignificantly lower PTSS than females<br><br>Age at diagnosis: Older age had insignificantly higher PTSS<br><br>*Previously reporting PTSS: Significantly higher PTSS (OR=1.19; p<0.01)	No relevant mediators studied and reported

Kwak et al. [35]	Up to 12 months after diagnosis	*Poorer prognosis of cancer: Significantly higher PTSS (p<0.001)<br><br>*Receiving active treatment at time of assessment: Significantly higher PTSS (p<0.01)<br><br>*Amongst patients receiving surgery, those who received surgery for staging reasons: Significantly lower PTSS than for treatment reasons (p<0.05)<br><br>*Experiencing more side effects: Significantly higher PTSS (p<0.001)	No relevant mediators studied and reported	Sex: Males had insignificantly higher PTSS than females<br><br>*Previously having experienced PTSS: Significantly higher PTSS (p<0.001)	*Experiencing disruption in employment / schooling: Significantly higher PTSS (p<0.05)

*Indicates variable studied had a significant association with the outcome (p-value <0.05).
Abbreviations: Odds ratio, OR; CI, confidence interval; Post-traumatic stress symptoms, PTSS.
Footnotes: Prikken et al, Jorngarden et al, Larsson et al and Kaplan et al are not included in this table as no relevant outcome mediators were studied and reported.

Table S3: Quality assessment of included cohort studies using the Joanna Brigg's Institute Critical Appraisal tool

Study	1	2	3	4	5	6	7	8	9	10	11	Total
Kunin-Batson et al. [26]	Y	Y	Y	N	N	NA	Y	Y	Y	Y	Y	8
Myers et al. [27]	Y	Y	Y	Y	Y	NA	Y	Y	Y	Y	Y	10
Werk et al. [32]	NA	Y	Y	Y	Y	NA	Y	Y	Y	Y	Y	9
Kaplan et al. [36]	Y	Y	Y	N	N	NA	Y	Y	Y	Y	Y	8
Desjardins et al. [33]	Y	Y	Y	N	N	NA	Y	Y	Y	Y	Y	8
Prikken et al. [34]	Y	Y	Y	N	N	NA	Y	Y	Y	Y	Y	8
Kwak et al. [35]	NA	Y	Y	Y	Y	NA	Y	Y	Y	Y	Y	9
Gupta et al. [38]	Y	Y	Y	N	N	NA	Y	Y	Y	Y	Y	8
Monteiro et al. [37]	Y	Y	Y	N	N	NA	Y	Y	Y	Y	Y	8
Sargin Yildirim et al. [28]	Y	Y	Y	Y	Y	NA	Y	Y	Y	Y	Y	10
Jorngarden et al. [29]	Y	Y	Y	N	N	NA	Y	Y	Y	Y	Y	8
Larsson et al. [30]	Y	Y	Y	Y	Y	NA	Y	Y	Y	Y	Y	10
Yardeni et al. [31]	Y	Y	Y	Y	U	NA	Y	Y	Y	Y	Y	9

Checklist
1. Were the two groups similar and recruited from the same population?
2. Were the exposures measured similarly to assign people to both exposed and unexposed groups?
3. Was the exposure measured in a valid and reliable way?
4. Were confounding factors identified?
5. Were strategies to deal with confounding factors stated?
6. Were the groups/participants free of the outcome at the start of the study (or at the moment of exposure)?
7. Were the outcomes measured in a valid and reliable way?
8. Was the follow up time reported and sufficient to be long enough for outcomes to occur?

9. Was follow up complete, and if not, were the reasons to loss to follow up described and explored?
10. Were strategies to address incomplete follow up utilized?
11. Was appropriate statistical analysis used?

Legend:

Y – Yes

N – No

U – Unclear

NA – Not applicable

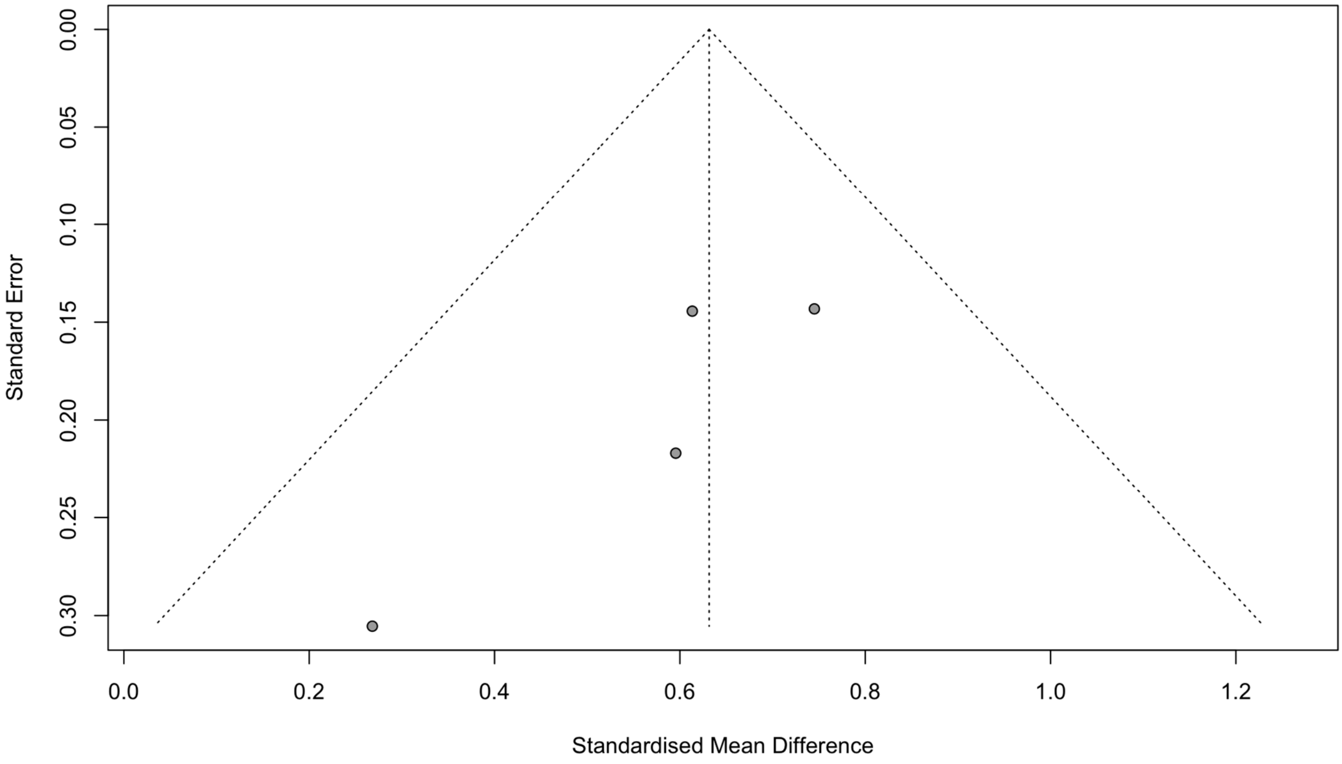

Figure S1: Funnel plot of baseline depressive symptoms.

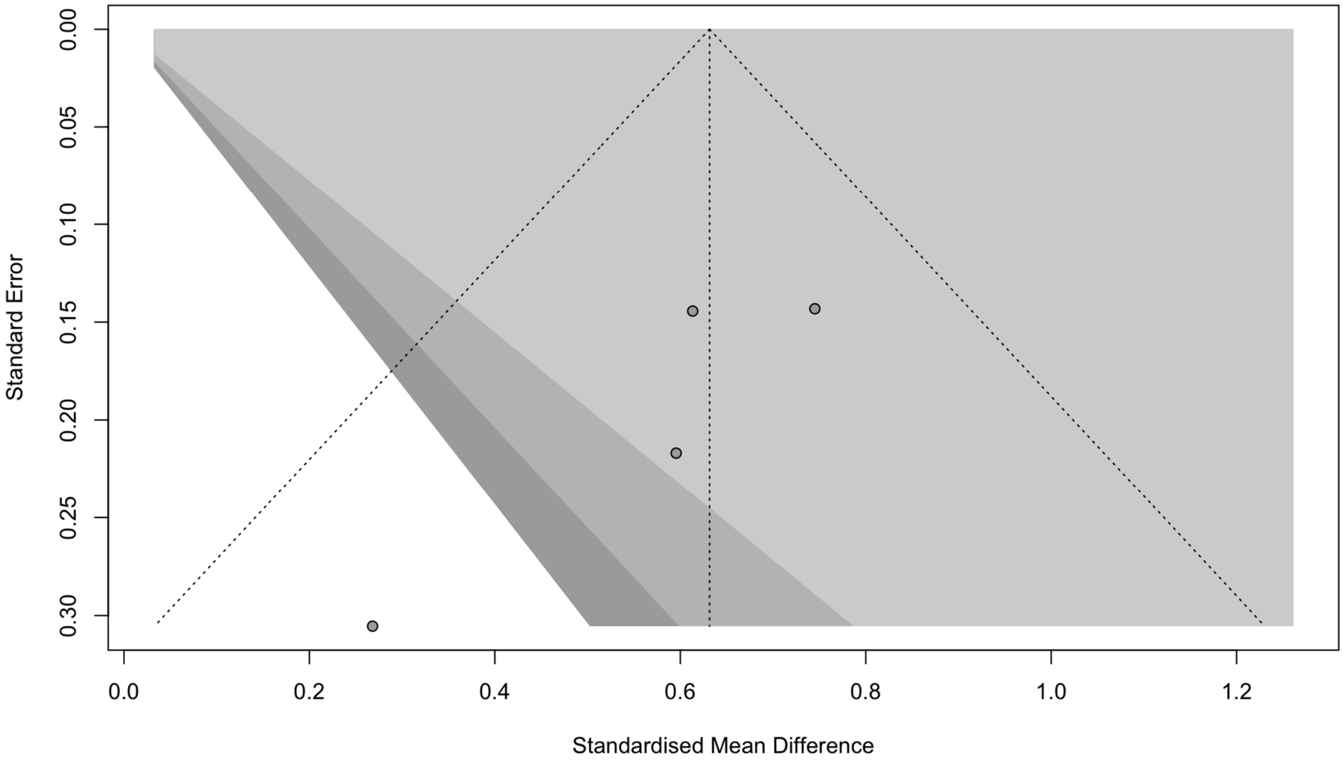

Figure S2: Trim-and-fill plot of baseline depressive symptoms.

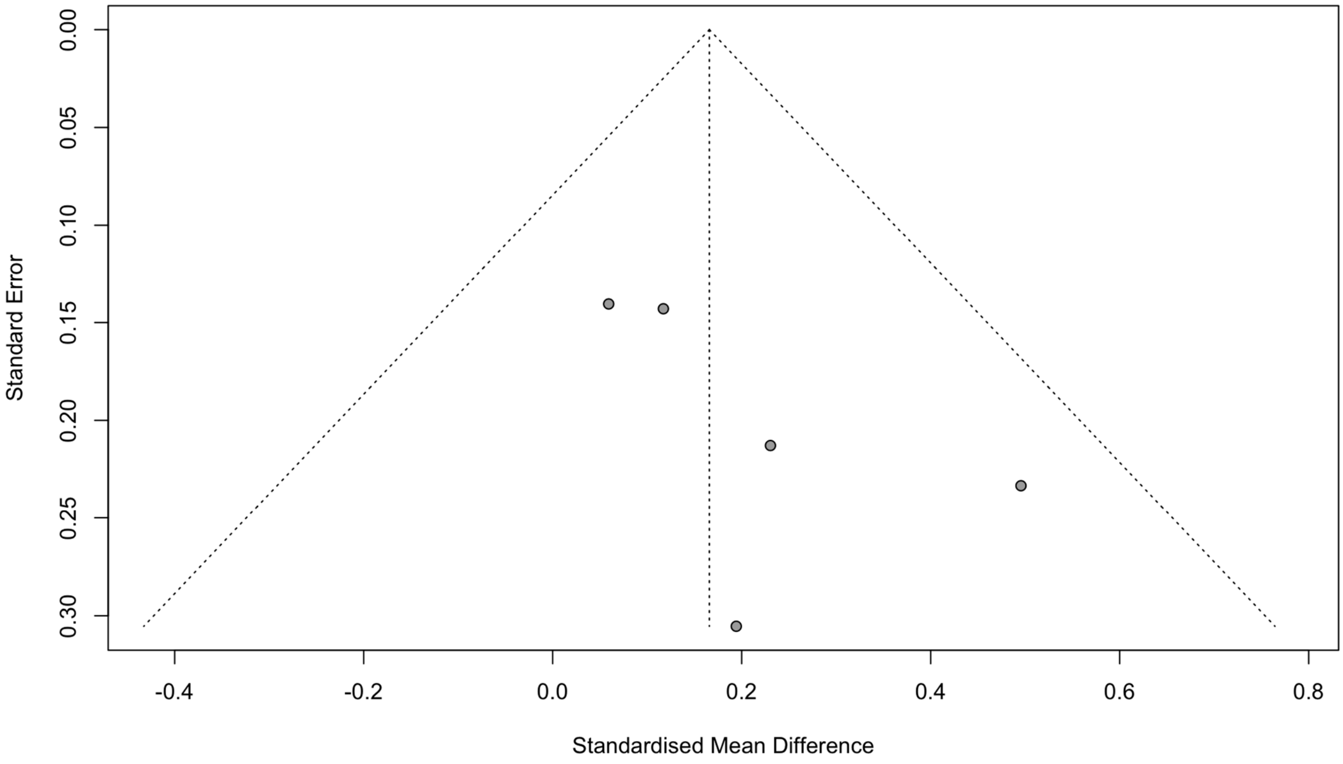

Figure S3: Funnel plot of baseline anxiety symptoms.

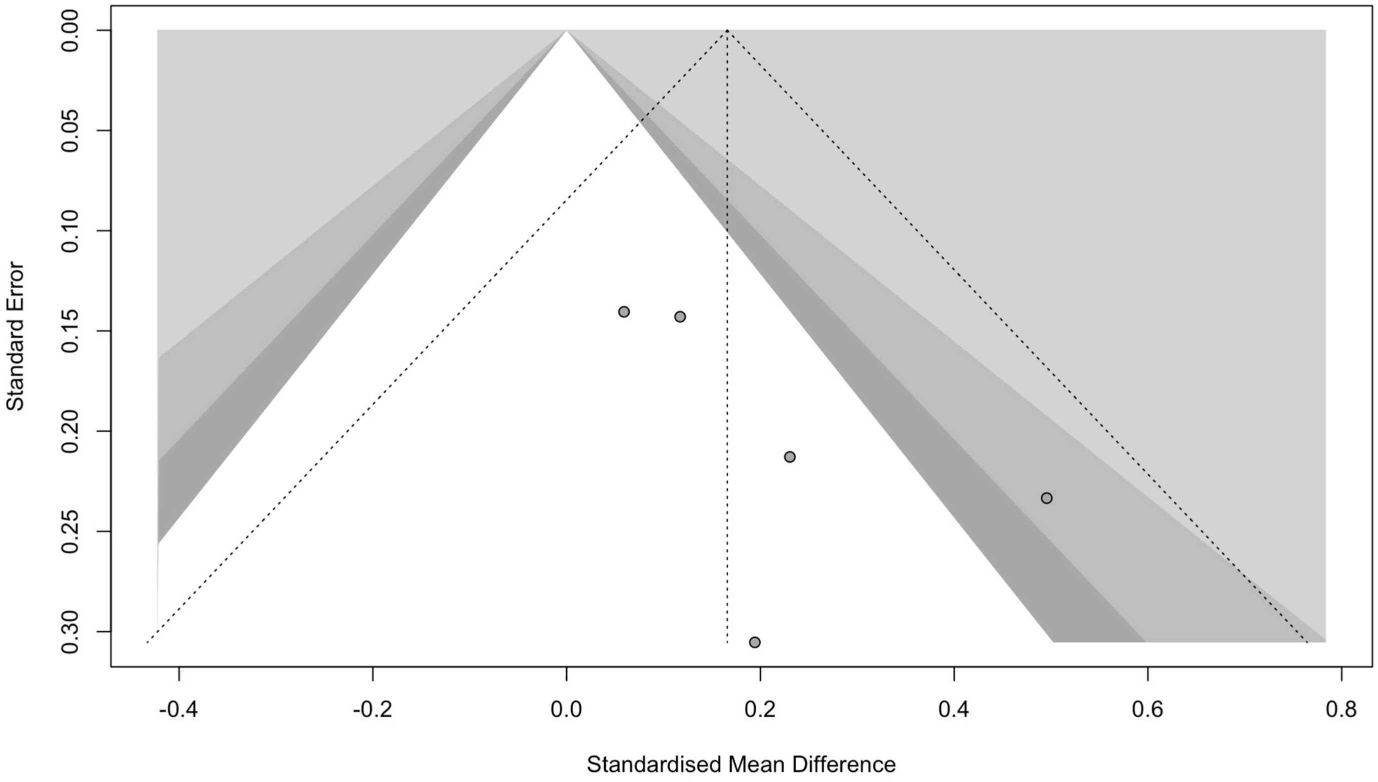

Figure S4: Trim-and-fill plot of baseline anxiety symptoms.
